# Intrahospital Handovers before and after the Implementation of ISBAR Communication: A Quality Improvement Study on ICU Nurses’ Handovers to General Medical Ward Nurses

**DOI:** 10.3390/nursrep14030154

**Published:** 2024-08-23

**Authors:** Marit Hegg Reime, Linda Skaug Tangvik, Mats Aleksander Kinn-Mikalsen, Tone Johnsgaard

**Affiliations:** 1 Department of Health and Caring Sciences, Faculty of Health and Social Sciences, Campus Bergen, Western Norway University of Applied Sciences, Inndalsveien 28, 5063 Bergen, Norway; tone.johnsgaard@hvl.no; 2 Lovisenberg Diaconal University College, Lovisenberggata 15B, 0456 Oslo, Norway; 3 Intensive and Postoperative Care Unit, Haraldsplass Diaconal Hospital, Ulriksdal 8, 5009 Bergen, Norway; linda.skaug.tangvik@haraldsplass.no; 4 Neonatal Intensive Care Unit (NICU), Østfold Hospital Kalnes, Kalnesveien 300, 1714 Grålum, Norway; mats.a.kinn-mikalsen@so-hf.no

**Keywords:** adherence, audit, general medical wards, intrahospital handover, intensive care unit, ISBAR, observational study, scoring tool

## Abstract

Background: Research finds a lack of structure as well as varying and incomplete content in intrahospital handovers. This study aimed to improve intrahospital handovers by implementing structured ISBAR communication (identification, situation, background, assessment and recommendation). Methods: This quality improvement study was conducted observing 25 handovers given by nurses from the intensive care unit to nurses from general medical wards at baseline and after the implementation of the ISBAR communication tool. The 26-item ISBAR scoring tool was used to audit the handovers. In addition, the structure of the ISBAR communication and time spent on the handovers were observed. Results: There were no significant improvements from baseline to post-intervention regarding adherence to the ISBAR communication scoring tool. The structure of the handovers improved from baseline to post-intervention (*p* = 0.047). The time spent on handovers declined from baseline to post-intervention, although not significantly. Conclusions: The items in the ISBAR communication scoring tool can act as a guide for details that need to be reported during intrahospital handovers to strengthen patient safety. Future research calls for studies measuring satisfaction among nurses regarding using different handover tools and studies using multifaceted training interventions.

## 1. Introduction

Clinical handovers are high-risk situations prone to communication failure and medical treatment errors [1,2,3,4,5]. Communication during patient handover is one of nine areas defined by the World Health Organization that can promote patient safety. The other eight are look-alike medication names, sound-alike medication names, patient identification, the performance of the correct procedure at the correct body site, the control of concentrated electrolyte solutions, assuring medication accuracy at transitions in care, avoiding catheter and tubing mis-connections, the single use of injection devices and improved hand hygiene to prevent healthcare-associated infection [6]. Handovers are defined as “the real time process of passing patient-specific information from one caregiver to another or from one team of caregivers to another for the purpose of ensuring the continuity and safety of a patient’s care” [7]. The Joint Commission asserts that 80% of adverse events involve miscommunication during handovers [8]. Contributing factors to ineffective handovers include time constraints, hierarchy, social barriers, lack of training in handover practices, lack of communication skills and lack of handover protocols [9,10]. Patient safety can be compromised if significant information is not passed on, leading to treatment delays, incorrect diagnoses, increased length of stay, erroneous treatment and lack of trust from patients [11,12,13]. For effective clinical handover to occur, face-to-face communication, adequate time, a common language and a standardised approach are all essential [14,15], as is formal training in this critical responsibility [13,16].

Research has found a lack of structure as well as varying and incomplete content in verbal intrahospital handovers, thus highlighting a need for more standardisation [17,18]. To increase patient safety in handover situations, the use of checklists is recommended [19,20]. Having originated in the US navy in the 1980s to ensure clear, precise communications between nuclear submarines and thereafter introduced into the US health service in 2001 [21,22], the ISBAR communication tool (identification, situation, background, assessment and recommendation) is recommended as the leading example of the standardisation of communication during patient handovers [23,24,25]. During identification (I), the persons initiating the handover introduce themselves and identify the patient by full name and identification number. Thereafter, the current patient situation in relation to treatment and care (S) is described, followed by the elaboration of the patient’s background (B), and at the end of the handover, an analysis of the situation is given (A) and advice on problems recommended (R) [26]. The ISBAR framework is an evidence-based approach providing the standardisation of handover communication, and the framework can be used in a wide range of clinical contexts, such as shift handover, patient transfer, interhospital transfers and the escalation of a deteriorating patient [22]. Although ISBAR is proving to be a valuable handover tool, for it to be successful, it must be effectively taught, and health professionals must be adequately trained in its use [22]. Research into the use of ISBAR in clinical practice has shown a potential 20% drop in the number of adverse events caused by communication failure [27], more effectively structured handover content [28,29], more prepared phone calls from nurses [30], improved quality of nursing care [31,32], increased clinical assessment and reasoning skills [30], increased interprofessional communication skills and confidence [33], more common expectations in content [26] and significantly improved handover quality in transfers between specialist wards [34]. Regarding the effectiveness in ISBAR implementation on patient outcomes, the evidence is still limited and connected to specific circumstances, such as communication over the telephone [4] or as one facet of other initiatives such as early warning system protocols, readmission risk assessments and daily interdisciplinary rounds, which may in itself have greater power to influence patient outcomes [35]. However, one does not know if the mixed results of ISBAR in patient outcomes may reflect problems with the implementation of the ISBAR tool [35].

Earlier studies have predominantly focused on intershift handovers, with relatively little attention given to intrahospital handovers [9,17]. Intershift handovers occur between care providers of the same department during shift changes. Handovers in intrahospital transfers occur when patients are transferred from one department to another during hospitalisation and presents challenges to those that are not experienced in intershift handoffs, such as tension between professions and different expectations regarding the content of the handover and the follow-up of patient care [9,36,37].

A systematic review has assessed the degree to which participants adhere to the use of ISBAR as intended [35]. Out of nine studies, four were performed in classroom settings and five in clinical settings. The studies varied in ISBAR training time, from 10 min to a full-day session, but they were typically 30–60 min. Three of the four studies of ISBAR implementation in classroom settings demonstrated large improvements in fidelity to ISBAR, whereas all five studies in clinical settings reported small to moderate improvements in fidelity [35].

Research has shown that ward nurses often feel overwhelmed by the information they receive from the intensive care unit and that they want more practical information regarding the patient’s status and follow-up plans [36,37,38]. Intrahospital handovers may require more collaborative and communicative interaction between different care units to ensure the continuity of patient care [16,39]. An umbrella review found that among nursing handover reviews, the settings included surgical and medical wards, intensive care wards and postoperative wards [40]. Nevertheless, intervention studies on intrahospital handovers between intensive care units and general medical hospital wards are still scarce [40,41,42].

## 2. Aim

Addressing research findings that intrahospital handovers lack structure as well as have varying and incomplete content, the aim of this study was to improve structured communication in intrahospital handovers using the ISBAR communication scoring tool as the audit tool [43]. The following research questions were asked, regarding whether there were improvements from baseline to post-intervention in the following: (1) adherence to the 26 items in the ISBAR scoring tool, (2) adherence to the structure of ISBAR communication and (3) time spent on the handover.

## 3. Materials and Methods

### 3.1. Design

This quality improvement study was conducted observing 25 handovers given by nurses from the intensive care unit to nurses from general medical wards at baseline and after the implementation of the ISBAR communication tool. This study aligns with recommendations from SQUIRE 2.0 (Standards for QUality Improvement Reporting Excellence) for Quality Improvement studies [44].

### 3.2. Quality Improvement Method

The science of improvement has its origins in the work of W. Edwards Deming [45], who stated that most services involve complex systems of interactions between healthcare professionals, procedures and equipment, organisational culture and patients. Quality improvement is a continuous process of identifying areas for improvement, testing interventions and adjusting them until the result is as desired and the improvement persists [46].

**Figure 1:**
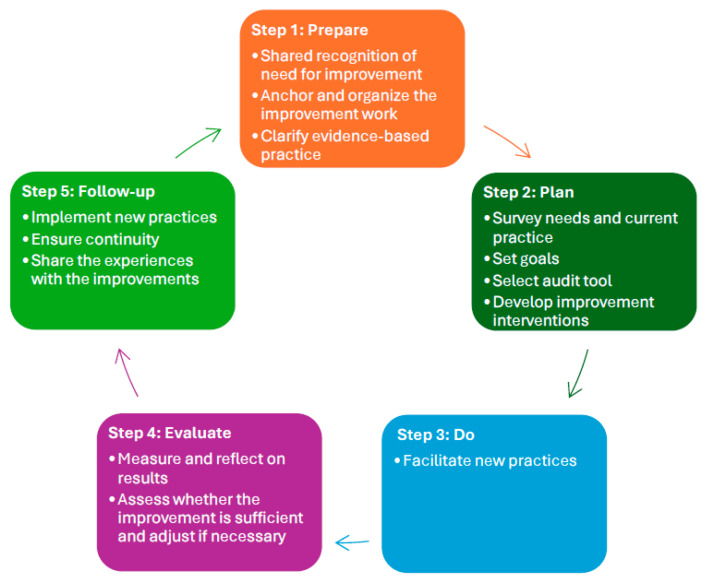
Model for quality improvement.

We used a five-phase model for quality improvement to guide our study. In this model, the various steps in the plan, do, study, act (PDSA) cycle are more specified [47]. This model can be used in both small and large improvement processes and can act as a to-do list for factors that research and experience have shown are necessary to ensure successful implementation [47] (Figure 1).

In the preparation phase, this study was anchored in the professional development department in the hospital. Since 2017, ISBAR has been a part of the Norwegian patient safety programme issued by the Directorate of Health [48] to give healthcare personnel a tool to ensure safe verbal communication. The professional development department recognised improvements in this area in the local hospital. Two of the members of the research team searched databases for evidence-based practice in handovers. For this quality improvement project, we narrowed this project to study handovers between intensive care units and general medical wards as this setting still represents a scarcity in research. Additionally, this enabled this project to be more easily managed. Due to not finding audit tools adapted to this context, an ISBAR communication scoring tool was developed in the planning phase [43]. Current practice was measured, and educational interventions were planned based on what was practically possible to achieve in busy clinical everyday life. In the third phase of the model, the educational interventions were carried out, followed by a post-intervention audit to evaluate if interventions were successful. Finally, the members in the professional development department reflected on the results and planned for new interventions which hopefully may contribute to improving the results.

### 3.3. Setting

The setting of this study was the transfer of intensive care patients with medical conditions from a level-1 ICU with 16 beds to three general medical wards with a total of 83 beds at one university hospital in Norway. This setting may be referred to as transferring patients from lifesaving care to rehabilitative care [49]. This study was carried out in 2018 as part of the hospital’s patient safety program. The nurses were not familiar with the ISBAR protocol prior to the intervention. The handovers took place in the nurses’ station at the general medical wards after transferring the patient from the intensive care unit.

### 3.4. Sample and Recruitment

The head nurse in the ICU informed the staff about this study on behalf of the researchers by distributing an information letter. Nurses who wanted to participate in this study and who fulfilled the inclusion criteria filled in a written consent form. The researchers than contacted the nurses to make further appointments for observation. A total of 30 nurses out of 60 available nurses in the intensive care unit signed the written consent form and were eligible for participation in this study. Due to practicalities, 25 nurses were observed at baseline and post-intervention. Of these, 17 were critical care nurses, 8 were nurses and 24 were female.

### 3.5. Inclusion Criteria

Intensive care nurses and nurses working more than a 50% position were included in this study. To be included in the post-intervention observation, the nurses should have completed the ISBAR intervention.

### 3.6. The ISBAR Intervention

We conducted 22 educational sessions between April and June 2018, each of 30 min duration, involving a total of 150 nurses from across the hospital. The sessions took the form of on-the-ward education, covering research regarding communication failures in the health service, how to use the ISBAR tool and the viewing of two videos: one not using the ISBAR communication tool and one using it, both with a duration of 90 s. In addition, pocket cards, mouse mats and posters that included all the relevant points in a handover were made available at the nurse’s station as reminder aids. The baseline observations were conducted in February and March, and the post-intervention observations were conducted in September and October.

### 3.7. Instrument

We developed and validated the ISBAR communication scoring tool [43] for use in intrahospital handovers between intensive care units and general medical wards (Table 1). The content of the tool was based on information needs in both kinds of departments based on expert review and previous research [9,36,38]. Seven critical care nurses with an average of 9 years’ work experience and seven nurses from medical wards with an average of 11 years’ work experience assessed the concurrent and content validity of the scoring tool. These nurses came from other hospitals and several different departments to avoid bias in the present study. The results from the validation process showed a CVI (content validity index) score of 0.92 for concurrent validity and an S-CVI score of 0.91 for the average calculation of the included items, indicating very good content validity [50].

In the present study, two observers with backgrounds as critical care nurses scored the intrahospital verbal handovers. For each item, a score of 1 was given if the item was included in the handover and a score of 0 if the item was not included. Both observers participated in 11 of the handovers at baseline to calculate inter-rater reliability. The inter-rater reliability was 91.6% and the Kappa score 0.86, indicating a very good inter-rater reliability [50]. The rest of the observations were therefore conducted separately by the two observers. In addition, field notes were taken to describe the structure of the handovers, and the time spent on the handovers were recorded.

### 3.8. Statistical Analysis

To compare baseline and post-intervention data, a chi-square test of independence and Fisher’s exact test were performed for proportions to calculate the *p*-value. Due to skewed distribution, the Mann–Whitney U test was used when comparing time spent on handovers at baseline and post-intervention. We were not always able to follow the same nurses at baseline and post-intervention due to practical reasons, which entailed the use of statistical tests for independent samples. To show the range of responses while excluding the most extreme, we present the 5th and 95th percentiles. A two-sided *p*-value of <0.05 was considered statistically significant. Data were analysed using IBM SPSS Statistics for Windows, Version 24.0 (Armonk, NY, USA: IBM Corp).

### 3.9. Ethical Considerations

This study was approved by the Norwegian Agency for Shared Services in Education and Research (no. 57512). Nurses from the intensive care unit signed a written declaration of consent to participate in this study. In addition, after reading the information letter, patients gave verbal consent to the nurse in charge to let the researchers observe the intrahospital handover. Fifty patients gave their verbal consent to participate, and one patient declined to participate. This study aligns with the principles in the declaration of Helsinki on voluntary participation, anonymity and confidentiality [51].

## 4. Results

### 4.1. Adherence to the 26 Items in the ISBAR Scoring Tool

There were no significant improvements from baseline to post-intervention in the 26-item ISBAR scoring tool (Table 1). Improvements were seen in 12 items (46%), impairments in 9 items (35%) and status quo in 5 items (19%).

Post-intervention, all handovers contained a summary of the given treatment (item 5) and information about the patient’s current condition (item 6). The greatest improvements were seen in stating the patient’s name (item 1) and stating the level of awareness (item 19), both with 16% improvement, followed by stating the year of birth (item 2), current condition (item 6), indicating significant psychological and cognitive state (item 11), intravenous access and treatment (item 18) and any treatment restrictions (item 26), with 12% improvement. The largest decline from baseline to post-intervention was seen in the stating of any infection and type of isolation (item 7) with a decline of 24%, stating any pain (item 22) and stating respiratory observations (item 15), with an 8% decline. The other items that declined did so by 4%.

**Table 1:** Baseline and post-intervention scores on the 26 ISBAR items.

Domain	ISBAR Items	Baseline (n = 25) N (%)	Post-Int (n = 25) N (%)	Difference	*p*-Value
Identification	1. State patients name	20 (80)	24 (96)	4 (16%)	0.189 ^b^
	2. State year of birth	20 (80)	23 (92)	3 (12%)	1.00 ^b^
Situation	3. State reason for admission	25 (100)	24 (96)	−1 (−4%)	1.00 ^b^
	4. State date of admission	23 (92)	22 (88)	−1 (−4%)	1.00 ^b^
	5. Give a brief summary of the treatment provided	23 (92)	25 (100)	2 (8%)	0.490 ^b^
	6. State current condition	22 (88)	25 (100)	3 (12%)	0.235 ^b^
	7. State any infection and type of isolation	10 (40)	4 (16)	−6 (−24%)	0.114 ^b^
Background	8. State previous illnesses of significance	23 (92)	22 (88)	−1 (−4%)	1.00 ^b^
	9. List medications of importance	22 (88)	21 (84)	−1 (−4%)	1.00 ^b^
	10. State allergy if relevant	1 (4)	1 (4)	0 (0%)	1.00 ^b^
	11. Indicate significant psychological and cognitive state	17 (68)	20 (80)	3 (12%)	0.333 ^a^
	12. State physical function level and mobilisation status	21 (84)	22 (88)	1 (4%)	1.00 ^b^
	13. State care needs and any dialogue with the municipality	16 (64)	16 (64)	0 (0%)	1.00 ^a^
	14. State special conditions for relatives (children < 18 years)	6 (24)	8 (32)	2 (8%)	0.529 ^a^
Assessment Airway Breathing	15. State observations (SaO_2_, RR, expectorate, blood gas)	22 (88)	20 (80)	−2 (−8%)	0.702 ^b^
	16. State treatment (O_2_ supplementation, inhalations, PEP/PEEP, pulmonary physiotherapy)	19 (76)	20 (80)	1 (4%)	0.733 ^a^
Circulation	17. State observations (BP, HR, temperature, diuresis)	23 (92)	23 (92)	0 (0%)	1.00 ^b^
	18. State treatment (IV accesses, IV treatment/infusion)	18 (72)	21 (84)	3 (12%)	0.306 ^a^
Disability	19. State observations of level of awareness (GCS/AVPU, pupils, MR/CT, NHISS score)	7 (28)	11 (44)	4 (16%)	0.239 ^a^
Exposure	20. State elimination status and any treatment	20 (80)	20 (80)	0 (0%)	1.00 ^a^
	21. State nutritional status (diet, nutrition score and treatment)	18 (72)	19 (76)	1 (4%)	0.747 ^a^
	22. State any pain (VAS and pain treatment)	11 (44)	9 (36)	−2 (−8%)	0.564 ^a^
	23. State any wounds or risk of wounds	9 (36)	8 (32)	−1 (−4%)	0.765 ^a^
	24. If not mentioned earlier, brief summary of examinations (performed/planned) or blood tests	21 (84)	20 (80)	−1 (−4%)	1.00 ^b^
Recommendation	25. Recommend follow-up treatment	22 (88)	22 (88)	0 (0%)	1.00 ^b^
	26. State any treatment restriction (CPR/respirator minus)	0 (0)	3 (12)	3 (12%)	0.235 ^b^

^a^ Chi-square test, ^b^ Fisher’s exact test. Abbreviations: SaO_2_ = oxygen saturation, RR = respiratory rate, PEP = positive expiratory pressure, PEEP = positive end-expiratory pressure, BP = blood pressure, HR = heart rate, IV = intravenous, GCS = Glasgow coma scale, AVPU = Alert–Verbal–Pain–Unresponsive, MR = magnetic resonance examination, CT = computed tomography examination, NHISS = National Institutes of Health Stroke Scale, VAS = visual analogue pain scale, CPR = cardiopulmonary resuscitation.

### 4.2. Adherence to the Structure of the ISBAR Communication

The observers’ field notes revealed that the intrahospital handovers were unstructured in 15 out of the 25 observations (60%) at baseline, compared to 8 of 25 observations (32%) in the post-intervention (*p* = 0.047). This improvement was particularly applicable to the clinical and physiological parameters under the assessment domain.

### 4.3. Time Spent on the Handover

No significant difference appeared between time spent on the handovers between baseline time and post-intervention time. However, the post-intervention median handover time declined by 61 s (Table 2).

## 5. Discussion

The aim of this study was to improve both the content and structure of ISBAR communication in intrahospital handovers when transferring patients from the intensive care unit to general medical wards. The results revealed that there were no significant improvements from baseline to post-intervention, which may be explained by the intervention not being effective, by a ceiling effect of initially high baseline scores, by different barriers, by the timeframe between baseline and post-intervention or by an underpowered study, which is likely in our study [50]. Barriers to guideline implementation can be differentiated into personal factors, guideline-related factors and external factors [52]. In our study, organisational changes at the hospital at the time around the implementation of ISBAR communication constituted the most challenging barrier since the hospital faced a major relocation process where all levels of the organisation were involved in the introduction of new technology. In retrospect, we see that it would have been more appropriate if the implementation phase had taken place at a quieter time. In addition, habits and routines in a communication process may be challenging to change, as well as nurses’ potential reluctance to learn how to use new tools, which may constitute personal barriers.

Our results are in line with the results from a systematic review that found small improvements in adherence to ISBAR communication in clinical settings compared to classroom settings [35]. On the other hand, field notes revealed that the structure of the intrahospital handovers improved significantly from baseline to post-intervention, which may be related to the intervention. Nevertheless, several handovers lacked important information about the patient’s condition, particularly in relation to the clinical and physiological parameters. This was a surprising finding, given that intensive care nurses are trained in the airway, breathing, circulation, disability, exposure (ABCDE) algorithm. If the receiving nurse does not obtain a good overview of the patient’s clinical condition on arrival, it may be more difficult to detect an early deterioration of the condition. This may affect how long it takes before necessary treatment is initiated, which in turn can have an impact on patient outcomes. A literature review by Elliott, Worrall-Carter and Page [53] revealed that ICU readmission rates ranged from 1.3% to 13.7%, primarily due to respiratory and cardiological complications and sepsis. The largest decline in our study was seen in the items state any infection and type of isolation, stating respiratory observations and stating any pain. However, we must bear in mind that among some of these items, the choice “not applicable” could be relevant for the respective patient, indicating that it is not a real decline. However, it is surprising that stating respiratory observations declined, as this is a significant part of vital signs within intensive care, as research has shown that the respiratory rate is the most sensitive marker showing patient deterioration but also a marker for showing improvements when it normalises [54]. Observational studies on ISBAR have revealed that the domains identification and situation are frequently reported, but background, assessment and recommendation are reported to varying degrees [42,55].

A national registry for adverse events in Norway found that 1459 patients started treatment too late [56]. The National Early Scoring Tool (NEWS) aims to prevent this by identifying early signs of changes in the patient’s condition [57,58]. At the time that the content validation of the ISBAR communication scoring tool was performed, the NEWS score was excluded due to a low I-CVI score, explained by the fact that these vital parameters are continuously monitored in intensive care patients. However, general wards have lower staffing levels and a reduced availability of resources contributing to challenges in caring for ICU survivors [59]. Thus, it will be useful to calculate the NEWS score before transferring patients to general ward care to have a starting point for whether the patient’s condition improves or worsens after arrival. Research also highlights the importance of measuring the NEWS score before transmitting patients from intensive care, as this score may predict clinical deterioration and readmission to intensive care departments [60,61,62]. Today’s intensive care patients are increasingly elderly and considered multimorbidity patients [63]. Our experience is that many of these patients have a documented decision to only receive restricted treatment by not initiating ventilator treatment or resuscitation in the event of a cardiac arrest. In our study, we found that treatment limitations were not stated in any of the handovers at baseline and in three handovers post-intervention. However, not knowing the patients’ medical records and the small sample size did not allow us to make any assumptions on this topic.

No significant difference was found regarding time spent on the handovers between baseline and post-intervention. However, the median post-intervention time declined by 61 s, which may be related to the handovers being better structured after the ISBAR intervention. This is also in line with studies by Cornell [64] and Uhm, Lim and Hyeong [65], indicating that ISBAR scoring tools do not further complicate the handover process. However, on the contrary, Petrovic et al. [66] found that the mean duration of the handovers increased by 2 min after implementing a handover protocol.

The ‘Swiss cheese model’ by James Reason [67] visualises how failures within a system can have fatal consequences if the ‘incident’ slips through all protective barriers. The ISBAR communication scoring tool may be a reminder which can seal gaps that may pose a threat to patient safety by improving both the content and structure of the handover. Research highlights that such standardised context-specific tools are useful in ensuring that all relevant information is communicated during clinical handovers [49,68,69], and a meta-analysis of standardised postoperative anaesthesia handovers demonstrated positive effects on provider, patient, organisational and handover outcomes [20]. Additionally, a Norwegian survey found that ward nurses felt more confident taking over the responsibility for patients after the implementation of ISBAR communication, and 95% of the nurses stated that ISBAR had improved patient treatment and patient safety [69]. However, there is a need for well-performed clinical trials to conclude that handover tools do in fact improve patient safety during the handover process from ICU to general wards [39].

## 6. Limitations

In retrospect, an extension of the educational session using simulation-based training may have strengthened the results, as a systematic review of systematic reviews supports such training in staff education [13]. However, training alone is often not sufficient to change practice. Given the complexity of changing communication behaviours in high-stakes clinical environments, potential impacts from an intervention comprising one 30 min education session seem limited. Future quality improvement studies might consider more intensive or multifaceted interventions, such as online learning, repeated sessions, role-play, simulation, workshops or ongoing feedback to effectively impact adherence to ISBAR communication [70]. Another limitation is that the observation items regarding the respiration and circulation domain contained several vital parameters with different measures, leaving the responsibility to the observers to judge if the information given in the handover was sufficient to give one point. Differentiating these items could have strengthened the instrument’s reliability. Another weakness was that it was difficult for the observers who did not know the patient to judge if an item was ‘not applicable’. Additionally, content validity is an important early step in instrument validation; however, further testing is needed, such as item analysis, factor analysis, internal consistency analysis and test–retest analysis.

The present study was conducted at one single site, limiting the results to be generalised to other hospitals. Another limitation is that the participants’ awareness of being observed produces behavioural changes and may lead to improved practice [50]. However, hidden observation was not applicable in the present study due to ethical principles of consent in research studies. Observer bias is also relevant in observational studies, where the expectations of the observers end up influencing the observations, such as the Rosenthal effect [50]. This may be an issue in intervention studies as researchers want the intervention to be successful, and in our study, the field notes on the structure of ISBAR communication may have such a bias. Tape recordings would have been useful as a quality check during the handovers, but tape recordings would have required additional approvals due to the storage of sensitive patient data. Nevertheless, observing ISBAR communication in clinical settings can reveal areas for improvement that would remain undiscovered by self-assessment alone.

The timeframe between baseline and post-intervention may also represent a limitation to this study, as one risks confounding factors such as other interventions or policy changes in the organisation. Additionally, if the timeframe is long, it might be more suitable to assess sustainability. Another limitation may be that the period between the implementation phase and the post-intervention also included the summer holidays, giving the participants less time to train in ISBAR communication.

## 7. Conclusions

There were no significant improvements from baseline to post-intervention regarding adherence to the 26-item ISBAR communication scoring tool. However, these items can act as a guide for details that need to be reported during a face-to-face intrahospital handover from ICU settings to general medical ward settings to strengthen patient safety. The structure of the handover improved significantly from baseline to the post-intervention phase. Time spent on handovers declined from baseline to post-intervention, although not significantly. Future research calls for studies measuring satisfaction among nurses when it comes to using different handover tools in addition to quality improvement projects using multifaceted training interventions. Robust studies measuring patient outcomes before and after the implementation of different handover tools are also in demand.

## Figures and Tables

**Table 2:** Time spent on handovers at baseline and post-intervention.

	Mean (Seconds)	Median (Seconds)	Percentiles (25–75%)	*p*-Value
Baseline	293	277	93–591	0.184 ^a^
Post-intervention	246	216	70–592	

^a^ Mann–Whitney U test.

## Data Availability

The original contributions presented in this study are included in this article; further inquiries can be directed to the corresponding author.

## Public Involvement Statement

There was no public involvement in any aspect of this research.

## Guidelines and Standards Statement

This study aligns with the SQUIRE 2.0 (Standards for QUality Improvement Reporting Excellence) for Quality Improvement studies [44].
